# Modulation of O-GlcNAcylation Regulates Autophagy in Cortical Astrocytes

**DOI:** 10.1155/2019/6279313

**Published:** 2019-11-13

**Authors:** Md. Ataur Rahman, Hongik Hwang, Yoonjeong Cho, Hyewhon Rhim

**Affiliations:** ^1^Center for Neuroscience, Korea Institute of Science and Technology (KIST), Seoul, Republic of Korea; ^2^Division of Bio-Medical Science and Technology, KIST School, Korea University of Science and Technology (UST), Seoul 02792, Republic of Korea

## Abstract

The addition of O-linked *β*-N-acetylglucosamine (O-GlcNAcylation) to serine and threonine residues is a common posttranslational modification of intracellular proteins which modulates protein functions and neurodegenerative diseases, controlled by a single pair of enzymes, O-GlcNAcase (OGA), and O-GlcNAcylation transferase (OGT). Autophagy is a cellular recycling pathway activated by stress and nutrient signaling; however, the mechanism by which O-GlcNAcylation modification regulates autophagy in cortical astrocytes is poorly understood. Here, we report that increased O-GlcNAcylation by the suppression of OGA activity using thiamet-G and OGA siRNA did not affect autophagy, whereas decreased O-GlcNAcylation caused by OGT inhibition by alloxan and OGT siRNA increased autophagy. OGT inhibitor and siRNA accumulated LC3 puncta, and cotreatment with chloroquine (CQ), an autophagy inhibitor, significantly increased LC3 puncta and LC3-II protein, confirming that decreased O-GlcNAcylation promotes autophagic flux. In particular, we found that OGT knockdown increases the fusion between autophagosomes as well as lysosomes and stimulates autophagy to promote lysosomal-associated membrane protein 1 (LAMP-1). Additionally, decreasing O-GlcNAcylation by treatment with alloxan, OGT siRNA, and OGA overexpression significantly decreased the level of autophagy substrate SQSTM1/p62, indicating that autophagic degradation was activated. Together, our study reveals a mechanism by which the modulation of O-GlcNAcylation modification regulates autophagy in mouse cortical astrocytes.

## 1. Introduction

O-linked-N-acetylglucosamine (O-GlcNAcylation), also called O-GlcNAcylation, is an abundant posttranslational modification of multiple proteins proposed to have both neurodegenerative as well as neuroprotective functions [1]. Previous studies indicate that O-GlcNAcylation regulates essential processes, such as metabolism [2], stress response [3], transcription [4], proteostasis [5], and autophagy [6]. O-GlcNAcylation transferase (OGT) covalently adds an O-GlcNAcylation moiety to serine or threonine residues of target proteins, while O-GlcNAcase (OGA) eliminates it [7]. A study of *Caenorhabditis elegans* suggests that increasing O-GlcNAcylation inhibits autophagy [6], whereas decreasing O-GlcNAcylation activates autophagic flux by mutant huntingtin protein [8]. Previous studies also reported that OGT is ubiquitously expressed and predominantly rich in the nucleus of neuronal cells [7] and synapses [2]. Importantly, the depletion or knockdown of OGT influences the fusion of autophagosomes with lysosomes and stimulates autophagic flux in mammalian cells [6]; OGT downregulation also effectively increases autophagy response to human bladder cancer cells [9]. Recently, it has been found that OGA inhibitors prompted autophagy in two mouse models of Alzheimer's disease (AD) as well as in primary neuron culture [10]. In addition, increasing O-GlcNAcylation levels by pharmacological inhibition of OGA decreases *β*-amyloid deposition, motor deficits, phosphorylation of tau, and impairments of memory function in certain mouse models of AD [11]. Despite the prevalence of O-GlcNAcylation modification, however, understanding the molecular basis of O-GlcNAcylation-regulated autophagic activity still requires further investigation.

Astrocytes, also known as astroglia, are star-shaped glial cells crucial for neuronal cell survival, development, biochemical support, repair and scarring process, synaptic connections, destroying pathogens and removing dead neurons, and homeostasis. Astrocytes comprise glutamate uptake which is dynamic for sustaining physiological stability as well as defending against neuronal toxicity [12]. It has been known that astrocytes are the only cells in the brain that accumulate and process cellular glycogen. Therefore, astrocytes contribute to influx glucose as well as energy intermediates into neuronal cells [13]. Astrocytes also contribute in blood-brain barrier development as well as conservation [14] and have an exclusive function in the clearance of brain solutes along veins [15]. However, astrocytes interconnect through each other and modulate the activity of neighboring cells mediated by a gap junction-combined network system which permits the direct cytoplasmic passage of ions in addition to small molecules [13, 16]. Furthermore, astrocytes possess receptors to a widespread range of peptides, neurotransmitters, cytokines, and hormones which control their own roles and also stimulate neurons and oligodendrocytes, as well as microglia [13, 16]. The physiological roles of astrocytes also play a key response in neurodegenerative progression and participate in the inflammatory function. Autophagy is usually considered as a degradative pathway, and in many neurodegenerative conditions, the autophagic function becomes inhibited. However, it has been found that autophagic dysregulation in astrocytes causes lysosomal storage disorders (LSDs) [17]. It is also determined that, like neurons, astrocytes play an important role in lysosomal storage as well as autophagy impairment through consequential accumulation of cytoplasmic protein aggregates in the cells. Therefore, astrocyte dysregulation alone could lead to neuronal degeneration and in turn can impact the pathological demonstration of neurodegeneration.

Autophagy is a cellular process that allows the degradation and recycling of dysfunctional and unnecessary cellular components and organelles in response to stress and starvation, as well as development [18]. During the autophagy process, a double-membraned vesicle called an autophagosome fuses with lysosomes, which is also known as autolysosomes, and the components are degraded and recycled by the process of autolysis [19]. Recently, it has been known that autophagy is closely associated with neurodegeneration [20], but very little is known about the relationship between O-GlcNAcylation and autophagy. It has been described that the transient OGA overexpression in Neuro2A cells upregulates autophagic flux by increasing the formation of autolysosomes [8]. Nonetheless, earlier studies examining the effect of O-GlcNAcylation in autophagy showed controversial results, and these discrepancies might be due to the difference in species, experimental design, or cell types. Moreover, the cellular function of O-GlcNAcylation modification throughout the autophagic progression *in vitro* and *in vivo* remains elusive. In the present study, we investigated how OGT inactivation increases autophagic activity via decreasing the O-GlcNAcylation level even though increasing the O-GlcNAcylation level by OGA inactivation does not affect autophagic activity in cortical astrocytes.

## 2. Materials and Methods

### 2.1. Reagents

Alloxan monohydrate, chloroquine diphosphate salt (CQ), and thiamet-G were obtained from Sigma-Aldrich (St. Louis, Missouri, United States). Anti-O-GlcNAcylation, anti-GFAP (GA5) Mouse mAb (Alexa Fluor® 488 Conjugate), anti-GFAP (GA5) Mouse mAb (Alexa Fluor® 555 Conjugate), LAMP-1, anti-LC3 (D3U4C) XP® Rabbit mAb (Alexa Fluor® 488 Conjugate), and anti-LC3 (D3U4C) XP® Rabbit mAb (Alexa Fluor® 555 Conjugate) antibodies were purchased from Cell Signaling Technology (Danvers, Massachusetts, United States). Anti-MGEA5 (OGA) and Anti-O-GlcNAcylation Transferase (OGT) antibodies were obtained from Proteintech Group Inc. (Chicago, Illinois, United States) and Sigma-Aldrich, respectively. OGA and OGT siRNAs were obtained from Santa Cruz Biotechnology, Inc. (Dallas, Texas, United States). LysoTracker® Green-HCK-123 was obtained from Molecular Probes Life Technologies Corporation (Eugene, Oregon, United States).

### 2.2. Cortical Astrocyte Culture

1-day-old (P1) ICR mice were used as the primary culture of astrocytes (Orient Bio Inc., Korea). Each brain was carefully isolated and separation was done inside Hank's buffered salt solution (HBSS) containing streptomycin and penicillin under a microscope. After careful removal of cerebral hemispheres, 0.1% trypsin-0.05% EDTA was used for digestion for 25 min at 37°C. Every 5 min, tissues were inverted. After 25 min, brain tissues were centrifuged at 1000 rpm for 3 min and supernatant was removed and then detrypsinized by DMEM medium. After that, cells were centrifuged at 1000 rpm for 3 min and washed with 1 ml fresh DMEM medium. Tissues were dissociated by Pasteur pipette size adjustment and centrifuged at 1000 rpm for 3 min. After supernatants were discarded, cells were seeded in a 100 mm culture dish in DMEM containing FBS (10%) and horse serum (10%) and grown at 37°C in 5% CO_2_. After 5 days, culture dishes were shaken manually to remove loosely attached neuronal cells and fresh medium was added. The astrocytes were used for further experiments.

### 2.3. Immunocytochemistry

After treatment and transfection, the astrocytes were washed with 1x ice-old PBS and fixed with methanol (100%) at -20°C for at least 15 min. After fixing, the astrocytes were washed 3 times with 1x PBS and blocked by 5% normal goat serum composed of 0.3% Triton™ X-100 in 1x PBS at 1 h. Cells were incubated with primary anti-GFAP conjugate with Alexa Fluor® 555 (1 : 50) and anti-LC3-II conjugate Alexa Fluor® 488 (1 : 50) in 1% BSA and 0.3% Triton™ X100 dissolved in 1x PBS overnight at 4°C. DAPI was added in 1x PBS for 10 min during washing time. LC3-II puncta were visualized and captured by a confocal microscope with the Leica Application Suite X (LAS X) (Leica Microsystems, Germany).

### 2.4. Autophagic Flux Counting

Puncta formation was counted and analyzed from the confocal image of immunocytochemistry analysis. At least 5 cells were counted from each image per condition, and the average number was plotted in a bar graph and results were presented via standard mean of error (±SEM). For measurements of autophagic flux *in vitro*, 10 *μ*M of the autophagosomal or lysosomal fusion inhibitor chloroquine (CQ) was used for 2 h before harvesting or fixing the cells and the autophagosomal marker anti-LC3-II conjugate Alexa Fluor® 488 was used for probing via an immunocytochemistry assay.

### 2.5. OGA and OGT Small Interfering RNA (siRNA) Transfection

Transient transfection was performed by siRNA of OGA (NCOAT siRNA (m): sc-62668; Santa Cruz Biotechnology, Inc.) and OGT (O-GlcNAc transferase siRNA (m): sc-40781; Santa Cruz Biotechnology, Inc.) with Lipofectamine™ 2000 according to the manufacturer's guidelines (Invitrogen; Carlsbad, California, United States). In detail, astrocytes were cultured in 6-well plates with 2 ml of DMEM without antibiotics. Lipofectamine™ 2000 was mixed and diluted with normal DMEM medium and kept at room temperature for 5 min. A siRNA oligomer with a final concentration of 50 nM was added with the Lipofectamine™ 2000-containing medium for 20 min. The complex mixture of siRNA and Lipofectamine™ 2000 was added on the astrocyte-containing medium and incubated for 48 h at 37°C. Scrambled siRNA with a final concentration of 50 nM was also added as control during the experiments. After a successful transfection, astrocytes were used for immunoblotting as well as immunocytochemistry studies.

### 2.6. Overexpression of Astrocytes by Adenovirus

Astrocytes were cultured in 12-well and 6-well dishes with 1 ml and 2 ml DMEM, respectively. Exogenous OGA (Mgea5) and OGT were overexpressed using adenoviruses, Ad-m-MGEA5/eGFP (ADV-264602, Vector Biolabs), and Ad-m-OGT/eGFP (ADV-266353, Vector Biolabs). 1 *μ*l and 2 *μ*l aliquots of Ad-m-MGEA5/eGFP and Ad-m-OGT/eGFP were added in 12-well and 6-well dishes for 48 h, respectively. After viral infection, the astrocyte cultures in 12-well dishes were used for immunocytochemistry and the cultures in 6-well dishes were used for immunoblotting analysis.

### 2.7. Dot Blot Analysis

To perform dot blot analysis, 1 *μ*l volume of each sample containing 1 *μ*g of proteins was spotted onto a polyvinylidene fluoride (PVDF) membrane. The membrane was dried in air for at least 30 min. After drying, the membrane was blocked in 5% BSA in 1x TBST for 1 h. After blocking, the spotted membrane was incubated with the Anti-O-GlcNAcylation Transferase antibody overnight at 4°C. The next day, the membrane was washed and subsequently incubated with the appropriate secondary antibody conjugate with horseradish peroxidase at room temperature for 1 h with gentle shaking. Washed three times with 1x TBST, the signal was detected using enhanced chemiluminescence (ECL) kits (iNtRON Biotechnology, Inc., Seoul, Korea). Protein bands were detected by the AlphaEase program (Alpha Innotech, San Leandro, California, United States). The band intensities were quantified with the ImageJ software.

### 2.8. Immunoblot Analysis

For immunoblotting, astrocytes were cultured on 6-well dishes. After drug treatment or transfection, astrocytes were harvested by radioimmunoprecipitation assay (RIPA) buffer (Elpis-Biotech. Inc., Daejeon, Korea). Collected cells were placed on ice for 30 min and centrifuged at 14,500 rpm for 10 min at 4°C. Collected proteins were quantified using Bradford (coomassie) protein assay kits (GenDEPOT, Katy, Texas, United States). 8-15% reducing gels were used depending on the protein size. In each well, an equal amount of proteins was loaded and separated by SDS-PAGE gel. After separation, proteins were transferred to a PVDF membrane. 5% skim milk or BSA was used for 1 h for blocking the transferred membrane. After washing with 1x PBST, the membrane was incubated overnight with the appropriate primary antibody at 4°C. The next day, the membrane was washed and treated with a secondary antibody conjugated with horseradish peroxide for a minimum of 2 h at room temperature. Finally, the membrane was washed three times and bands were detected using ECL kits using the AlphaEase program.

### 2.9. Statistical Analysis

All the statistical analyses were performed using GraphPad Prism (San Diego, California, United States). Results are indicated as mean ± SEM, and statistical significance was determined by an unpaired Student *t*-test. *p* < 0.05 was considered as a significant value of these studies.

## 3. Results

### 3.1. Pharmacological Modulation of O-GlcNAcylation Regulates Autophagy in Mouse Cortical Astrocytes

**Figure 1:**
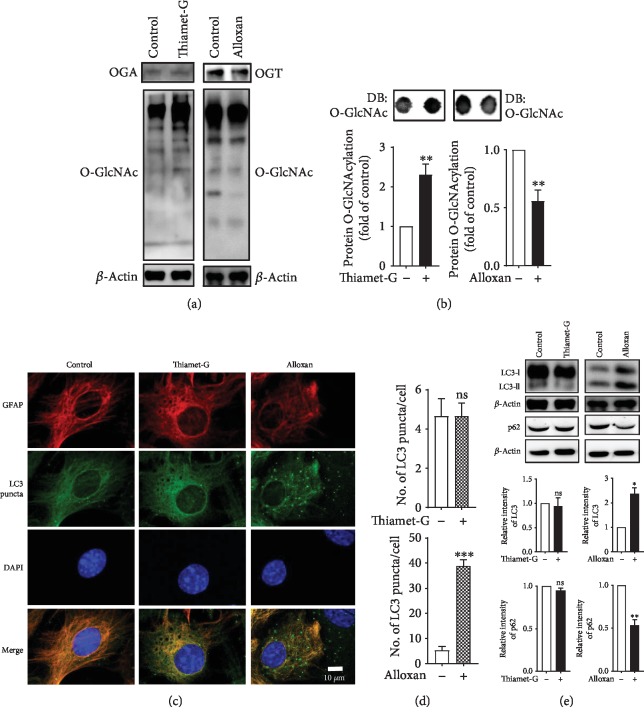
Pharmacological inhibition of OGA and OGT modulates autophagy in cortical astrocytes. (a) Immunoblot analysis of OGA, OGT, and O-GlcNAcylation levels in astrocytes treated with thiamet-G (1 *μ*M) or alloxan (5 mM) for 24 h. (b) Dot blot analysis of O-GlcNAcylation levels after thiamet-G or alloxan treatment for 24 h. Densitometry quantification of O-GlcNAcylation protein was performed by ImageJ. All statistical analyses were obtained as ±SEM (*n* = 3, ^∗∗^*p* < 0.01). (c) Autophagic flux in astrocytes was visualized by immunofluorescence staining with anti-GFAP (red) and anti-LC3 (green) antibodies using confocal microscopy. (d) LC3 puncta formation was measured from at least 3 independent random areas from each slide, and the puncta from a minimum of 5 cells which contained them were counted and calculated (ns = nonsignificant, ^∗∗∗^*p* < 0.001). (e) Immunoblot analysis of LC3 and p62 levels in astrocytes treated with thiamet-G or alloxan (*n* = 3, ns = nonsignificant, ^∗^*p* < 0.05, ^∗∗^*p* < 0.01).

To understand the process of autophagy, it is important to know the molecular role of microtubule-associated protein 1 light chain 3 (LC3) which is a ubiquitin-like modifier protein generally involved in autophagosome biogenesis in autophagy signaling. In the initial autophagy process during phagophore membrane formation, pro-LC3 is progressed to form LC3-I, which is successively conjugated to phosphatidylethanolamine (PE) to make LC3-II, where it stimulates the formation of an autophagosome. Through the subsequent combination of autophagosomes and lysosomes, intravacuolar LC3-II, which is known as an autolysosome, is generally degraded. Therefore, the formation as well as the degradation of LC3-II is a valuable and useful conventional established method for the measurement and monitoring of the autophagy process [21]. Additionally, sequestosome 1, also known as SQSTM1/p62, is a multifunctional signaling molecule used as an indicator of autophagy and is involved in a diversity of cell signaling pathways. SQSTM1 is one of the well-known substrates of autophagy, and thus it is extensively used as an important indicator of autophagic degradation. SQSTM1 is primarily degraded by autophagy [22]. In the current study, we selected LC3-II as an autophagy marker due to its abundance within the brain [23] and SQSTM1/p62 autophagic substrates which are eventually degraded. As a first step to assess whether modulating O-GlcNAcylation regulates the autophagy pathway in cortical astrocytes, we pharmacologically controlled the O-GlcNAcylation level and determined the effects by immunoblot and immunocytochemistry assay. To elucidate the modulation of O-GlcNAcylation, we treated the cells with thiamet-G [24] and alloxan [25], inhibitors of O-GlcNAcase (OGA) and O-GlcNAc transferase (OGT), respectively, and found that thiamet-G treatment significantly increased O-GlcNAcylation, whereas alloxan-treated cells significantly reduced O-GlcNAcylation examined via immunoblot and dot blot analysis (Figures 1(a) and 1(b)). However, immunofluorescence staining with an anti-LC3 antibody showed that the number of LC3 puncta per cells was not changed by thiamet-G even dose-dependently (supplementary [Supplementary-material supplementary-material-1]), whereas alloxan-treated astrocytes significantly increased LC3 puncta (Figures 1(c) and 1(d)). In addition, confirming the autophagic regulation of O-GlcNAcylation modulation, we observed that thiamet-G-treated cells did not significantly alter LC3-II and p62 protein expression (Figure 1(e)), whereas alloxan-treated cells significantly increased LC3-II and decreased p62 expression in cortical astrocytes (Figure 1(e)). These data together indicate that reducing the O-GlcNAcylation level promotes autophagy in cortical astrocytes.

### 3.2. OGT Inhibition Enhances Autophagic Flux in Cortical Astrocytes

**Figure 2:**
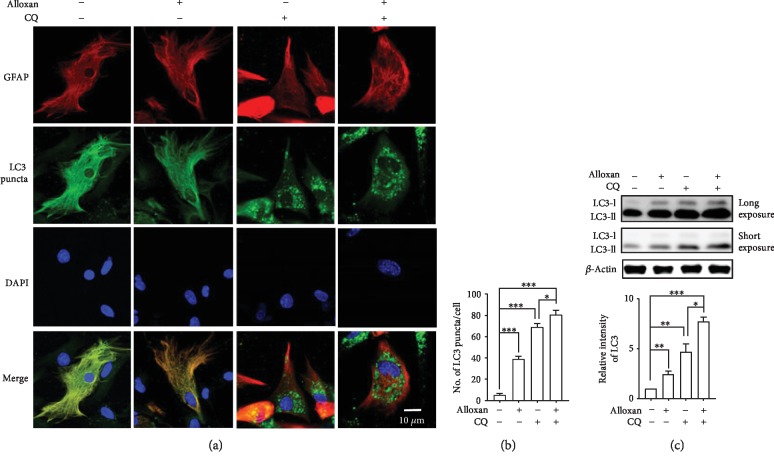
Inhibition of OGT by alloxan enhances autophagic flux in astrocytes. (a) Autophagic flux in astrocytes was examined by immunofluorescence staining with the astrocyte-specific markers anti-GFAP (red) and anti-LC3 (green) using a confocal microscope. CQ was treated before 2 h of fixation. (b) Counting and calculation of LC3 puncta formation (^∗^*p* < 0.05, ^∗∗∗^*p* < 0.001). (c) Immunoblotting of LC3 proteins was performed in cells treated with alloxan and CQ, and statistical analyses were performed by ±SEM (*n* = 3, ^∗^*p* < 0.05, ^∗∗^*p* < 0.01, and ^∗∗∗^*p* < 0.001).

To understand how O-GlcNAcylation modulation influences autophagy, we investigate whether an OGT inhibitor regulates autophagic flux activity determined by an LC3 turnover assay [21]. In this method, cells are treated with chloroquine (CQ) and ammonium chloride (NH_4_Cl), lysosomotropic agents which are also known as autophagy inhibitors, which protect the acidification of lysosomes or inhibit the fusion between autophagosomes and lysosomes, therefore preventing the degradation of LC3-II as a result of the increasing levels of LC3-II [26]. Thus, the alteration of LC3-II during drug treatment and the presence or absence of an autophagy inhibitor actually measures the amount of LC3-II delivered to the lysosomes for degradation by the autophagy process [27]. To study whether alloxan regulates autophagic flux activity or not, we first analyzed astrocytes in the absence and presence of the autophagy inhibitor CQ [28]. We found that CQ significantly increased the amount of LC3 puncta and LC3-II expression compared to the control. However, to investigate autophagy regulated by O-GlcNAcylation, we evaluated an OGT inhibitor which reduces the O-GlcNAcylation level as well as stimulates autophagy. Earlier, we found that the OGT inhibitor alloxan significantly increased LC3 puncta and LC3-II expression (Figure 1); here, we observed that cotreatment with CQ and alloxan further upregulated LC3 puncta formation compared to CQ treatment alone (Figures 2(a) and 2(b)). Immunoblot analysis also confirmed that cotreatment with alloxan and CQ further increased the amount of LC3-II expression (Figure 2(c)). Thus, this observation suggests that decreasing O-GlcNAcylation significantly upregulates autophagic flux in cortical astrocytes.

### 3.3. Knockdown of OGA and OGT Modulates O-GlcNAcylation and Controls Autophagy in Cortical Astrocytes

**Figure 3:**
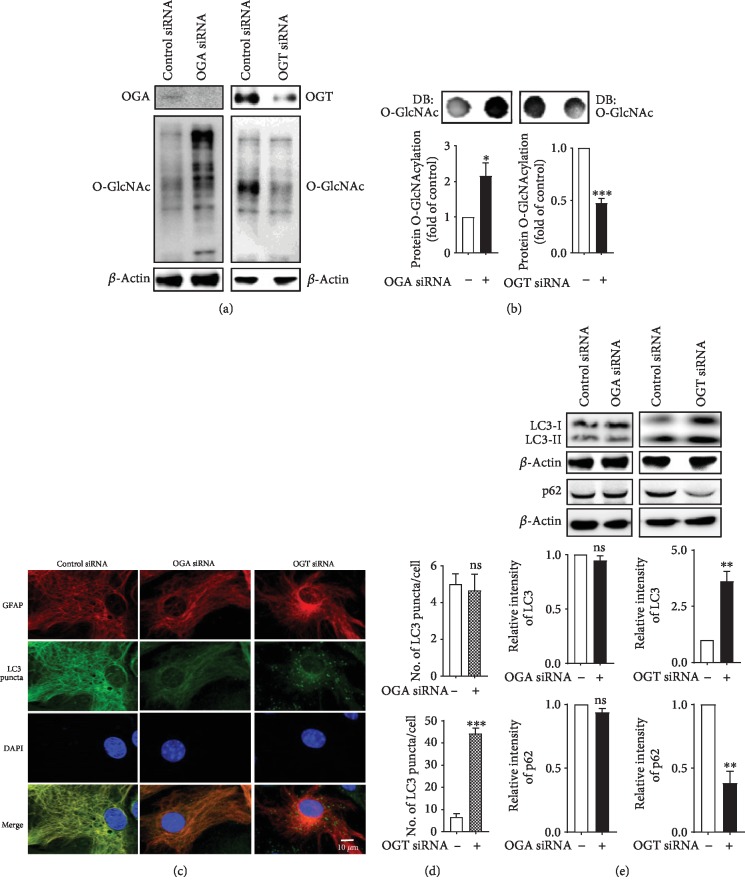
Effects of OGA and OGT knockdown on autophagy in astrocytes. (a) Astrocytes were transfected with OGA and OGT siRNA for 48 h. Immunoblot showing OGA, OGT, and O-GlcNAcylation levels. (b) Dot blot analysis of O-GlcNAcylation in cells transfected with OGA or OGT siRNA (*n* = 3, ^∗^*p* < 0.05, ^∗∗∗^*p* < 0.001). (c) Immunofluorescence staining of astrocytes was performed by astrocyte-specific anti-GFAP and anti-LC3 antibodies via confocal microscopy. (d) The number of LC3 puncta was counted and analyzed by ±SEM (^∗∗∗^*p* < 0.001, ns = nonsignificant). (e) Immunoblot was indicated by LC3 and p62 expression of OGA- or OGT siRNA-treated cells (*n* = 3, ^∗∗^*p* < 0.01).

We also examined whether the genetic manipulation of O-GlcNAcylation levels displays any diverse effects in astrocytes. A previous study showed that the reduction of O-GlcNAcylation caused by OGT regulates autophagic activity [29], but only a limited number of studies have examined the effects in brain cells. To investigate whether O-GlcNAcylation regulates autophagic activity, we genetically modulate O-GlcNAcylation levels by transfecting cultured cortical astrocytes with control and short interfering RNA (siRNA) targeting OGA and OGT. After the transfection of OGA and OGT siRNA, both of these proteins were decreased and the levels of O-GlcNAcylations were up- and downregulated in cortical astrocytes, respectively (Figure 3(a)). The change in O-GlcNAcylation levels was also confirmed by dot blot analysis (Figure 3(b)). However, to study the autophagic effects, we found that OGA siRNA did not change LC3 puncta formation, but OGT siRNA significantly increased puncta formation using anti-LC3 immunofluorescence staining (Figures 3(c) and 3(d)). In addition, immunoblot analysis also shows that LC3-II and p62 expressions were not significantly altered by OGA siRNA, while OGT siRNA significantly increased LC3-II and decreased p62 intensity (Figure 3(e)). Therefore, this investigation suggests that genetic manipulation of O-GlcNAcylation might regulate autophagy in cortical astrocytes.

### 3.4. Knockdown of OGT Increases Autophagic Flux in Cortical Astrocytes

**Figure 4:**
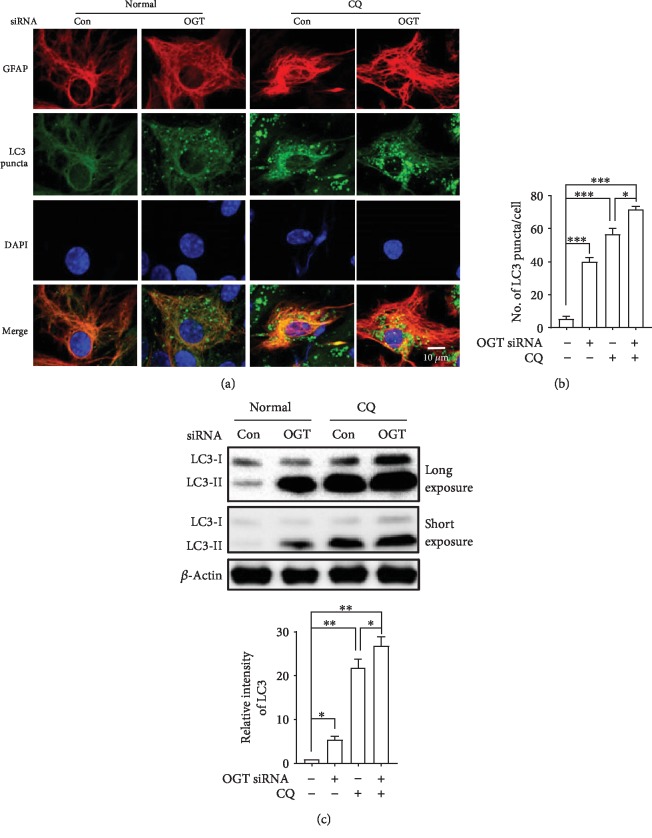
Knockdown of OGT enhances autophagic flux in astrocytes. (a) Autophagic flux was observed by immunofluorescence with anti-GFAP and anti-LC3. CQ was treated 2 h prior to the fixation of cells. (b) Statistical analyses of LC3 puncta were plotted by ±SEM (^∗∗^*p* < 0.01, ^∗∗∗^*p* < 0.001). (c) LC3 levels were determined in OGT-transfected and CQ-treated cells via immunoblot and plotted via ±SEM (*n* = 3, ^∗^*p* < 0.05, ^∗∗^*p* < 0.01).

To address whether OGA knockdown influences autophagy in astrocytes, we used the LC3 turnover assay. Here, we performed LC3-specific immunofluorescence staining after cotreatment with the autophagy inhibitor CQ, and the number of puncta per cells was significantly increased by CQ treatment. We performed LC3 turnover in OGT siRNA-transfected cells. The number of LC3 puncta was significantly increased by the inhibition of the autophagy inhibitor CQ, and interestingly, cotreatment with CQ and OGT siRNA further elevated the number of LC3 puncta per cells as revealed by immunofluorescence staining (Figures 4(a) and 4(b)). Using immunoblot analysis, we further confirmed that LC3-II was significantly increased with the cotreatment with CQ and OGT siRNA in cortical astrocytes (Figure 4(c)). Together, these findings indicate that reduction of O-GlcNAcylation modification by OGT inactivation increases autophagic flux in cortical astrocytes.

### 3.5. OGT Knockdown Promotes Interaction and Fusion of Autophagosomes and Lysosomes in Cortical Astrocytes

**Figure 5:**
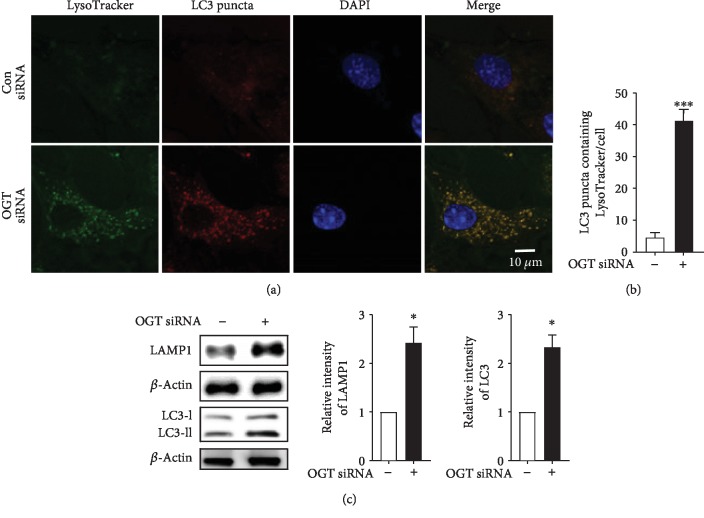
OGT knockdown promotes fusion between autophagosomes and lysosomes in astrocytes. (a) After OGT siRNA transfection, LysoTracker® Green-HCK-123 was treated and placed at 37°C for 2 h prior to fixation. Immunofluorescence staining was done by anti-LC3 antibody, and imaging was done via confocal microscopy. From each slide, 3 random areas were selected from which an image could be taken. (b) The number of LC3 puncta containing LysoTracker was counted from at least 5 cells from each slide (^∗∗∗^*p* < 0.001). (c) LAMP-1 and LC3 expression levels were quantified by immunoblot (*n* = 3, ^∗^*p* < 0.05).

In mammalian cells, the fusion of autophagosomes and lysosomes is important to complete the overall autophagic flux by the association of lysosomal proteins [30]. To study the role of autophagosomes binding to lysosomes, we examined the lysosome detection marker LysoTracker, a green-fluorescent dye which tracks and labels in the acidic organelles of living cells. In the present study, we found that OGT siRNA-transfected cells show that the number of LC3 puncta containing LysoTracker was significantly increased in cortical astrocytes as revealed by double immunofluorescence staining of LC3 and the LysoTracker marker (Figures 5(a) and 5(b)). However, to confirm the enrolment of lysosomal fusion with autophagosomes, we also examined lysosomal-associated membrane protein 1 (LAMP-1) and LC3-II protein via immunoblot analysis. The results from our investigation indicated that the relative expression levels of LAMP-1 and LC3-II were significantly unregulated by OGT siRNA-transfected cells (Figure 5(c)). We performed a time-dependent assay using chloroquine (CQ) in OGT siRNA-transfected cells to inhibit autophagosome-lysosome fusion. Here, we found that CQ did not time-dependently show any remarkable change in LC3-II, LysoTracker, and LAMP-1 expression (Figures [Supplementary-material supplementary-material-1] and [Supplementary-material supplementary-material-1]). Additionally, we checked whether by decreasing O-GlcNAcylation by alloxan treatment, OGT siRNA, and OGA overexpression, the level of lysosomal activity is increased ([Supplementary-material supplementary-material-1]). Thus, this study indicates that OGT knockdown induces autophagy through the fusion of lysosomes in cortical astrocytes.

### 3.6. Modulation of O-GlcNAcylation through OGA and OGT Overexpression Regulates Autophagy in Astrocytes

**Figure 6:**
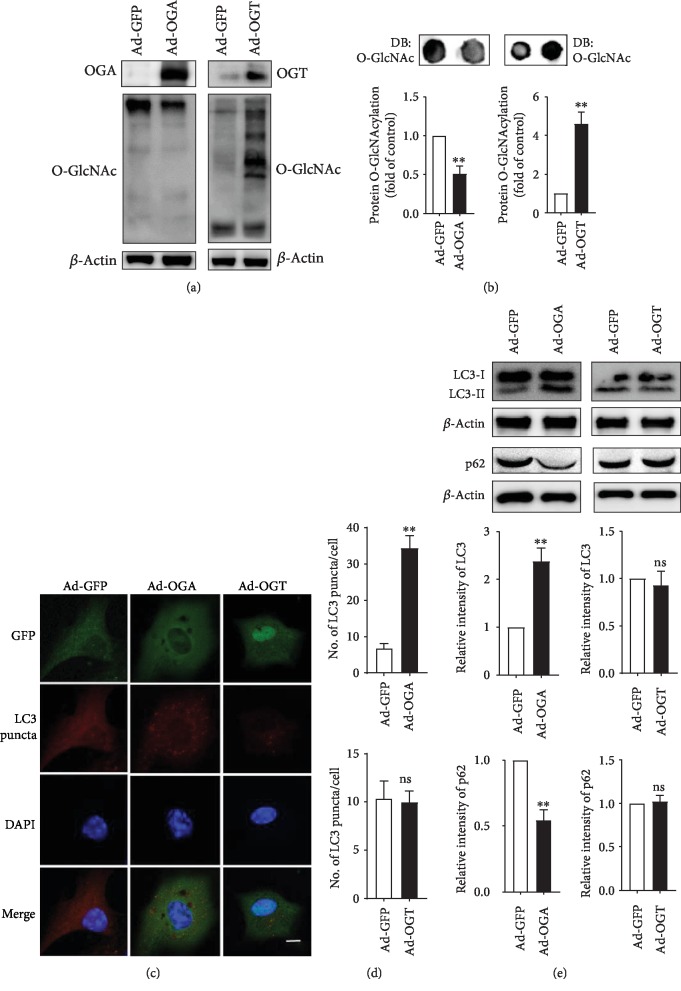
Overexpression of OGA or OGT regulates autophagy in astrocytes. (a, b) OGA or OGT was overexpressed by Ad-m-MGEA5/eGFP or Ad-m-OGT/eGFP for 48 h, respectively. Overall O-GlcNAcylation levels were examined by dot blot and analysis by ±SEM (^∗^*p* < 0.05). (c) Immunofluorescence staining was prepared using anti-GFAP and anti-LC3 antibodies. (d) The number of LC3 puncta was determined by analysis by ±SEM (^∗^*p* < 0.05, ns = nonsignificant). (e) LC3 and p62 expression levels were determined by immunoblotting after overexpression of Ad-OGA or Ad-OGT and analyzed by ±SEM (*n* = 3, ^∗∗^*p* < 0.01, ns = nonsignificant).

The O-GlcNAcylation cycle is regulated by OGA and OGT, but their expression is also controlled by OGA and OGT overexpression. Both the O-GlcNAcylation cycle and autophagy are highly conserved stress-activated pathways as well as nutrient signaling pathways; therefore, O-GlcNAcylation cycling may be involved in autophagy regulation via the modulation of O-GlcNAcylation through the overexpression of OGA and OGT [20]. To further elucidate the role of O-GlcNAcylation modification in autophagy regulation, we overexpressed OGA or OGT using an adenovirus in cortical astrocytes. Immunoblot analysis revealed that both OGA and OGT proteins were upregulated by the overexpression of these enzymes, and OGA overexpression significantly downregulated O-GlcNAcylation (Figure 6(a)). On the other hand, the adenoviral-mediated overexpression of OGT increased OGT protein and O-GlcNAcylation expression (Figure 6(b)). A reduction of O-GlcNAcylation by ad-OGA significantly increased the number of LC3 puncta cells, whereas enhancing O-GlcNAcylation by OGT overexpression did not show increasing puncta formation (Figures 6(c) and 6(d)). Lastly, western blot revealed that the levels of the autophagy marker LC3-II and the p62 substrate were significantly increased and decreased by ad-OGA, respectively, and there were no significantly changed LC3-II and p62 levels by ad-OGT overexpression (Figure 6(e)). Together, these results strongly indicate that O-GlcNAcylation cycle regulation plays a key role in controlling autophagy in cortical astrocytes.

### 3.7. Overexpression of OGA Promotes Autophagic Flux in Cortical Astrocytes

**Figure 7:**
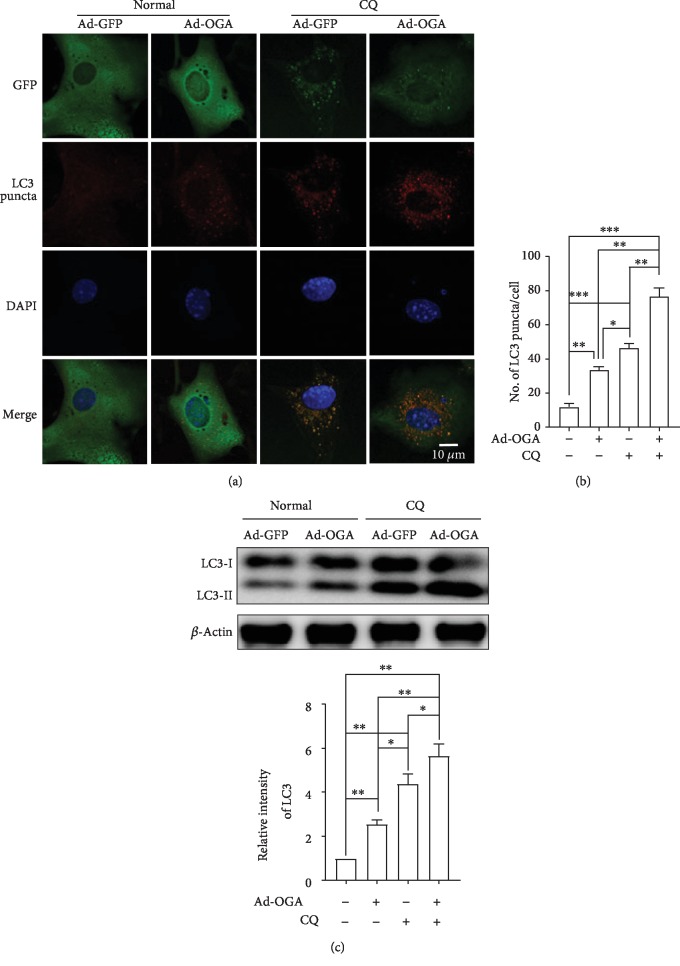
OGA overexpression promotes autophagic flux in cortical astrocytes. (a) Astrocytes were transfected by Ad/eGFP and Ad-m-MGEA5/eGFP adenovirus for 48 h. Representative immunofluorescence was examined by anti-GFP and anti-LC3 antibodies. (b) Statistical analysis was calculated by counting the number of LC3 puncta-containing cells. Significance of different conditions was analyzed through ±SEM (^∗^*p* < 0.05, ^∗∗^*p* < 0.01, and ^∗∗∗^*p* < 0.001). (c) LC3 protein expressions were revealed by immunoblotting after overexpression of control and ad-OGA. Cells were treated with CQ before 2 h of cell lysates (*n* = 3, ^∗^*p* < 0.05, ^∗∗^*p* < 0.01).

To determine whether OGA and OGT overexpression regulates autophagic activities, we checked the autophagic flux measured by LC3 turnover assays. OGA overexpression alone and the treatment with an autophagy inhibitor CQ alone significantly increased LC3 expression. Interestingly, however, cotreatment with ad-OGA and CQ further enhanced the number of LC3 puncta formation when examined through LC3 immunofluorescence (Figures 7(a) and 7(b)). In addition, immunoblot analysis also confirmed that enhanced LC3-II protein expression was observed under cotreatment with ad-OGA and CQ, suggesting that decreasing O-GlcNAcylation has the capacity to elevate autophagic flux (Figure 7(c)). Taken together, these results also clearly suggest that O-GlcNAcylation modulation by OGA overexpression is an important factor to regulate autophagy in cortical astrocytes.

## 4. Discussion

To understand the molecular function of O-GlcNAcylation-mediated autophagy regulation *in vitro*, increasing evidence suggests that suppressing O-GlcNAcylation enhances autophagic activity. Earlier it has been reported that the modification of O-GlcNAcylation via OGT knockdown stimulates autophagic flux in mammalian cells as well as in *C. elegans* [6]. However, the actual role of O-GlcNAcylation modification on autophagy has not been identified, and it is not clear why OGA and OGT overexpression or knockdown modulates autophagy, given that these two enzymes have shown opposite functions. To recognize the function of O-GlcNAcylation in autophagy, we used mouse cortical astrocytes as an *in vitro* model. Herein, our data showed that OGT was the target of GlcNAcylation modification in astrocytes and the attenuation of O-GlcNAcylation by OGT inactivation promotes autophagy activation in cortical astrocytes.

Preceding studies have revealed that perturbations in overall O-GlcNAcylation levels have contradictory effects on autophagy in several animal models, cells, and peripheral tissues [6, 31–33]. However, many autophagy enhancers, including rapamycin and trehalose, reduce toxic tau species and amyloid-*β* toxicity in mouse models of tauopathy as well as in APPswe/PS1deltaE9 and are responsible for the protective benefits of autophagy [34, 35]. The main hypothesis of these studies has been described here as the enhancement of autophagy via the modulation O-GlcNAcylation levels in brain cells. Therefore, to confirm our hypothesis, we tested two pharmacologically widely used O-GlcNAcylation modulators, thiamet-G and alloxan, to regulate autophagy. The data from our investigation indicates that OGA inhibition increases O-GlcNAcylation but does not affect autophagic activity (Figures 1 and 2). On the other hand, autophagic activity is significantly increased when the O-GlcNAcylation level was reduced by an OGT inhibitor (Figures 1 and 2). Therefore, these collective data suggest that the inhibition of O-GlcNAcylation is important to stimulate autophagy in cortical astrocytes.

Both O-GlcNAcylation and autophagy are extremely conserved with stress-activated and nutrient signaling pathways; however, only limited studies have examined the probability that O-GlcNAcylation might be implicated in regulating the basic autophagy pathway in the brain. It has been exposed that some key autophagic proteins such as Beclin-1, in addition to Bcl-2, are important targets of O-GlcNAcylation modification which impairs autophagic pathways in a diabetic heart [32]. In contrast, mutations of the O-GlcNAcylation cycle control enzymes in *C. elegans* leading to elevated LC3 protein expression during starvation conditions [31, 36]. From our observations, OGT knockdown considerably increases autophagic flux via reducing the level of O-GlcNAcylation in astrocytes, although the knockdown of OGA does not show autophagic activity (Figures 3 and 4). Furthermore, a recent study found that O-GlcNAcylation also hinders the autophagic activity through reducing Atg protein levels in addition to autophagosome formation in the fly model of *Drosophila melanogaster* [33]. Therefore, the current studies strongly suggest that O-GlcNAcylation cycling might have a role to regulate autophagy at several stages.

In addition, the formation of autophagosomes, which eventually fuse with lysosomes, is important to complete the degradation and reuse of the vesicular contents in mammalian cells [37]. It has been described that reducing O-GlcNAcylation improves the accumulation of autophagosomes and lysosomes, thus increasing autophagic activities which are associated with the reduction of mHtt aggregation in fly models as well [8]. In particular, O-GlcNAcylation of the SNARE protein SNAP29 inhibits autophagic flux and autophagosome-lysosome fusion in association with autophagy inhibition [6]. However, the specific interaction between O-GlcNAcylation modification and autophagy regulation remains unknown. We showed that OGT knockdown remarkably stimulates the fusion between autophagosomes and lysosomes determined by LysoTracker staining (Figure 5). A similar observation was seen in the knockdown of OGT that promotes both autophagosome formation as well as maturation [6]. Besides, in tauopathy models, O-GlcNAcylation shows a harmful function against the mutation of huntingtin toxicity in fly models as well as in cells [8]. Eventually, our investigations also explain that OGA overexpresses reduced O-GlcNAcylation and increases autophagy and significantly enhances autophagic flux in cortical astrocytes (Figures 6 and 7). Therefore, these findings suggest that the O-GlcNAcylation cycle is important to regulate autophagy in brain cells.

In conclusion, it has been well studied that the stimulation of autophagy acts with beneficial effects in a number of neurodegenerative diseases [38, 39]. However, the mutation of the OGT function stimulates basal autophagic activity and autophagy enhancement may directly impact on the clearance of aggregate and abnormally misfolded proteins such as amyloid *β*, tau protein, and dysfunctional organelles comprising senile plaques in the cell [6, 40, 41]. Therefore, we predict that targeting OGT inhibition might help to defend against a wide variety of neurodegenerative diseases by stimulating autophagy.

## Data Availability

The raw data used to support the findings of this study are available from the corresponding author upon request.

## Supplementary Materials

Supplementary Materials Figure S1: dose-dependent effects of thiamet-G on astrocytes. (A) Autophagic flux by immunofluorescence staining with anti-GFAP (red) and anti-LC3 (green) antibodies is shown via confocal microscopy. (B) Thiamet-G-treated LC3 expression levels are shown by immunoblotting. Figure S2: time-dependent effects of CQ on OGT knockdown astrocytes are shown by immunoblotting LC3 and LAMP-1 expressions. Figure S3: time-dependent studies of CQ in OGT-transfected astrocytes by LysoTracker® Green-HCK-123 (green) and anti-LC3 puncta (red) imaging are shown via confocal microscopy. Figure S4: alloxan, OGT siRNA, and OGA overexpression on astrocytes examined by LysoTracker® Green-HCK-123 and anti-LC3 puncta (red) immunofluorescence staining is shown via confocal microscopy.
