# Supplementary material for: Modulation of O-GlcNAcylation Regulates Autophagy in Cortical Astrocytes
Source: Oxid Med Cell Longev. 2019 Nov 13;2019:6279313. doi: 10.1155/2019/6279313 (PMC6881589; doi:10.1155/2019/6279313)

**Supplementary Information**

**Modulation of O-GlcNAcylation regulates autophagy in cortical astrocytes**

Md. Ataur Rahman^1^, Hongik Hwang^1^, Yoonjeong Cho^1, 2^, Hyewhon Rhim^1, 2*^

^1^Center for Neuroscience, Korea Institute of Science and Technology (KIST), Seoul, Republic of Korea; ^2^Division of Bio-Medical Science and Technology, KIST School, Korea University of Science and Technology (UST), Seoul 02792, Republic of Korea.

**Running title:** O-GlcNAcylation modulates autophagic activity.

***Correspondence should be addressed to:** Hyewhon Rhim, Center for Neuroscience, Brain Science Institute, Korea Institute of Science and Technology (KIST), 5 Hwarang-ro 14-gil, Seongbuk-gu, Seoul 02792*,* Republic of Korea. Tel.: +82-2-958-5923, Fax: +82-2- 958-6937. E-mail: [hrhim@kist.re.kr](mailto:hrhim@kist.re.kr)

**Supplementary methods**

**Immunocytochemistry**

After treatment and transfection, astrocytes were washed with 1X ice cold PBS and fixed with methanol (100%) at -20°C at least 15 min. After fixing, washed 3 times through 1X PBS and blocked by 5% normal goat serum compose of 0.3% Triton™ X-100 in 1X PBS at 1 h. Cells were incubated with primary anti-GFAP conjugate with Alexa Fluor® 555 (1:50) and anti-LC3-II conjugate Alexa Fluor^®^ 488 (1:50) in 1% BSA and 0.3% Triton™ X100 dissolve in 1X PBS overnight at 4°C. DAPI was added in 1X PBS for 10 min during washing time. LC3-II puncta were visualized and captured by confocal microscopy of Leica Application Suite X (LAS X) (Leica Microsystems, Germany).

**Immunoblot analysis**

For immunoblotting, astrocytes were cultured on 6-well dishes. After drug treatment or transfection, astrocytes were harvested by radioimmunoprecipitation assay (RIPA) buffer (ELPIS-BIOTECH. Inc., Daejeon, Korea). Collected cells were placed on ice for 30 min, and centrifuged 14,500 rpm 10 min at 4°C. Collected proteins were quantified using Bradford (coomassie) protein assay kits (GenDEPOT, Katy, Texas, United States). 8-15% reducing gels were used dependent on the protein size. In each well equal amount of proteins were loaded and separated by SDS-PAGE gel. After separation proteins were transferred to a PVDF membrane. 5% skim milk or BSA for 1 h was used for blocking of transferred membrane. After washing with 1X PBST, membrane was incubated with appropriated primary antibody for overnight at 4°C. Next day, membrane was washed and treated with secondary antibody conjugated with horseradish peroxide minimum 2 h at room temperature. Finally, three times washed and bands were detected using ECL kits using AlphaEase program.

**Supplementary figure legends**

**Figure S1.** Dose-dependent effects of thiamet-G on astrocytes. (A) Astrocytes were treated 1, 10, and 30 µM of thiamet-G for 24h. Autophagic flux in astrocytes were visualized by immunofluorescence staining with anti-GFAP (red) and anti-LC3 (green) antibodies using a confocal microscopy. (B) After thiamet-G treated by indicated doses, LC3 expression levels were determined by immunoblotting.

**Figure S2.** CQ time-dependent effects of OGT knockdown astrocytes. (A) Astrocytes were transfected with control and OGT siRNA for 48 h. CQ (10 µM) was treated 1h, 2h, and 3h prior to harvest. Representative immunoblot shown LC3 and LAMP-1 expression in different time point of CQ treatment.

**Figure S3.** Time-dependent studies of CQ in OGT transfected astrocytes. After OGT siRNA transfection, CQ (10 µM) was treated for 1 h, 2 h, and 3 h. LysoTracker^®^ Green-HCK-123 was treated and placed at 37°C for 2 h prior to fixation. Immunofluorescence staining was done by anti-LC3 antibody and imaging via confocal microscopy. From each slide 3 random areas were selected to take image.

**Figure S4.** Effects of alloxan, OGT siRNA, and OGA overexpression on astrocytes. LysoTracker^®^ Green-HCK-123 was treated and placed at 37°C for 2 h prior to fixation. Immunofluorescence staining was done by anti-LC3 antibody and imaging via confocal microscopy.

**Supplementary figures**

**Figure S1.**

**
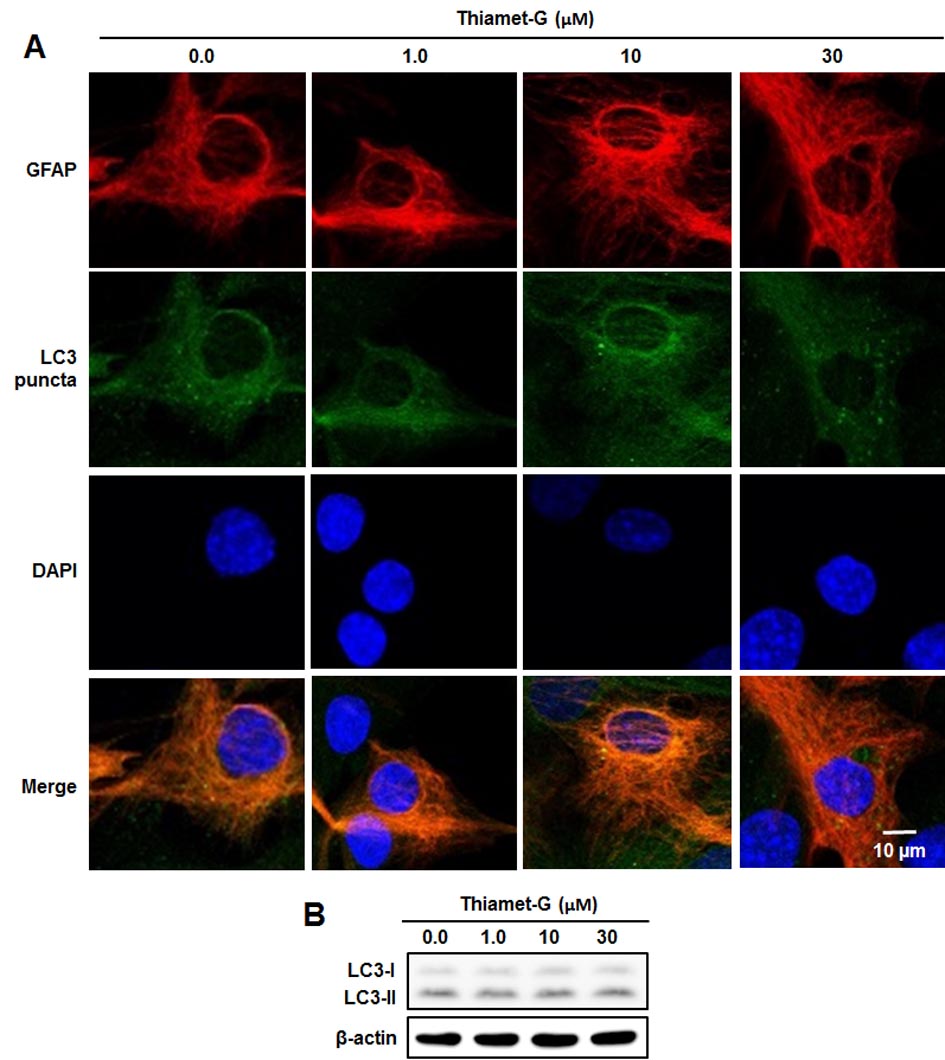
**

**Figure S2.**

**
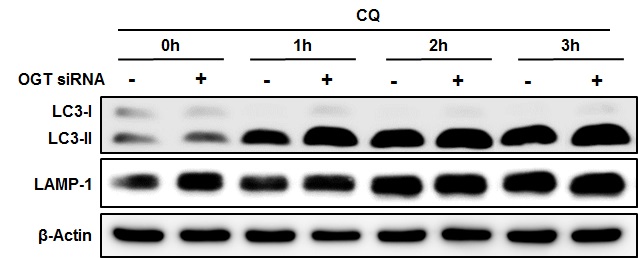
**

**Figure S3.**

**
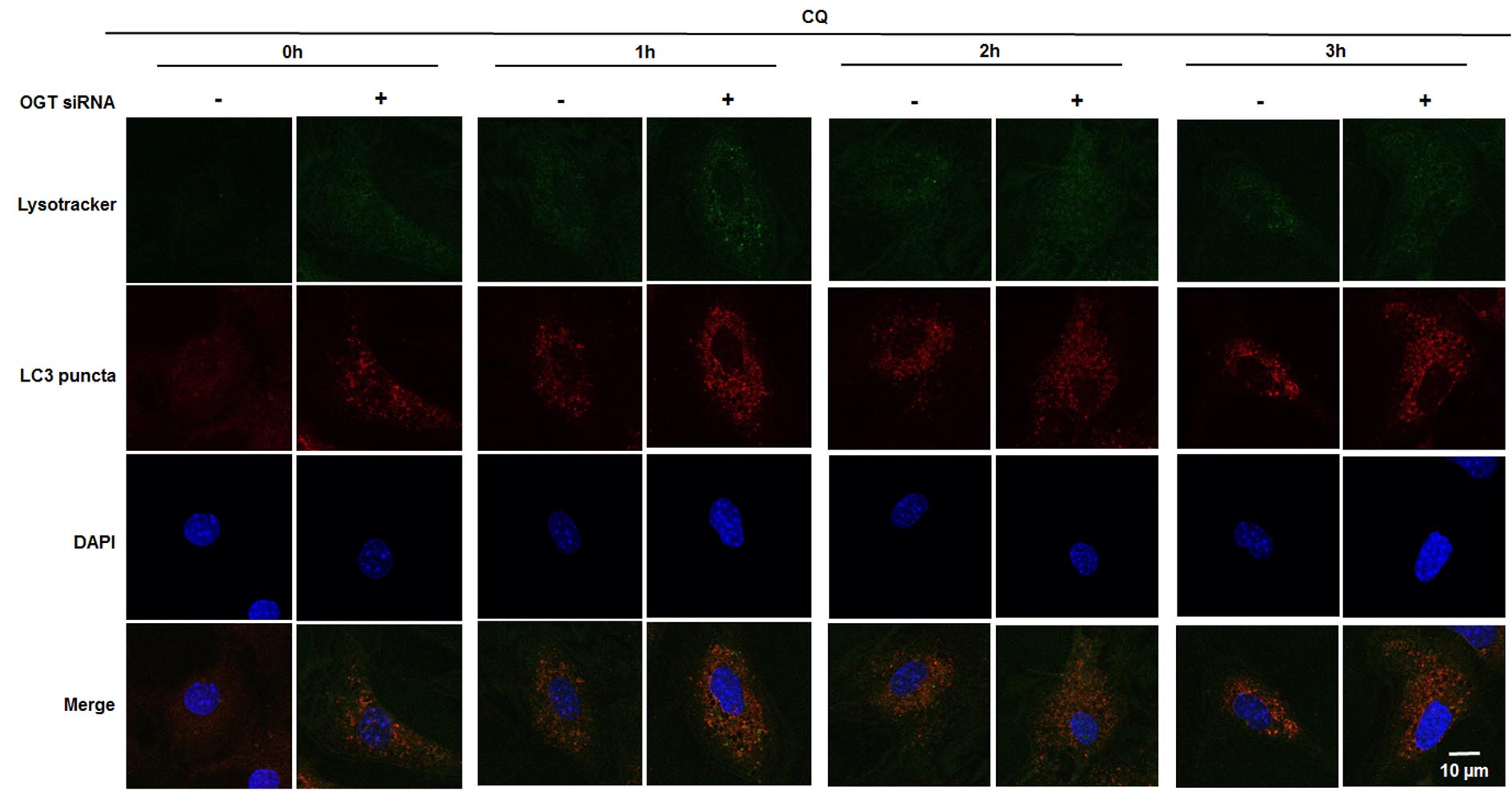
**

**Figure S4.**


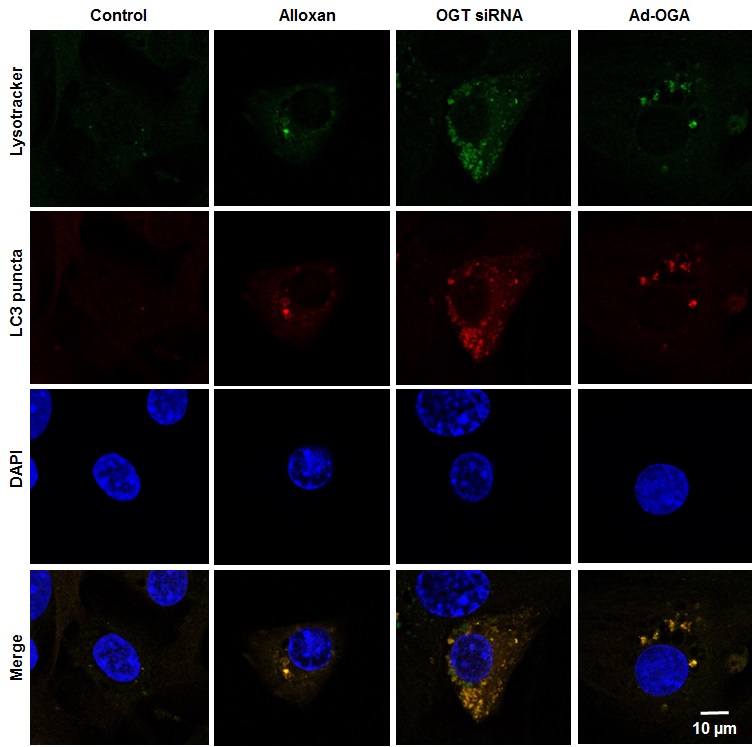

Supplement: Supplementary Materials — Figure S1: dose-dependent effects of thiamet-G on astrocytes. (A) Autophagic flux by immunofluorescence staining with anti-GFAP (red) and anti-LC3 (green) antibodies is shown via confocal microscopy. (B) Thiamet-G-treated LC3 expression levels are shown by immunoblotting. Figure S2: time-dependent effects of CQ on OGT knockdown astrocytes are shown by immunoblotting LC3 and LAMP-1 expressions. Figure S3: time-dependent studies of CQ in OGT-transfected astrocytes by LysoTracker® Green-HCK-123 (green) and anti-LC3 puncta (red) imaging are shown via confocal microscopy. Figure S4: alloxan, OGT siRNA, and OGA overexpression on astrocytes examined by LysoTracker® Green-HCK-123 and anti-LC3 puncta (red) immunofluorescence staining is shown via confocal microscopy. [file 6279313.f1.docx]
